# Chronic Kidney Disease in Non-Diabetic Older Adults: Associated Roles of the Metabolic Syndrome, Inflammation, and Insulin Resistance

**DOI:** 10.1371/journal.pone.0139369

**Published:** 2015-10-02

**Authors:** Andrea R. Zammit, Mindy J. Katz, Carol Derby, Markus Bitzer, Richard B. Lipton

**Affiliations:** 1 Saul B. Korey Department of Neurology, Albert Einstein College of Medicine, Bronx, New Yourk, United States of America; 2 Einstein Aging Study, Albert Einstein College of Medicine, Bronx, New York, United States of America; 3 Department of Epidemiology and Population Health, Albert Einstein College of Medicine, Bronx, New York, United States of America; 4 Department of Internal Medicine, University of Michigan, Ann Arbor, Michigan, United States of America; University of Leicester, UNITED KINGDOM

## Abstract

**Background:**

The aims of the study were to examine the association between CKD and the metabolic syndrome (MetS) and its components in older adults. We also explored two possible pathways linking the metabolic syndrome with CKD: inflammation as measured by high sensitivity C-Reactive Protein (hsCRP) and insulin resistance as measured by HOMA-IR.

**Methods:**

Community-dwelling non-diabetic 70+ adults from the Einstein Aging Study participated in the study. We defined CKD as eGFR below 60mL/min/1.73m^2^. MetS was defined according to recent guidelines from the National Cholesterol Education Program. Binary logistic regressions were used to assess the association between the metabolic syndrome, its components and CKD with adjustments for demographics, HOMA-IR and hsCRP.

**Results:**

Of 616 participants (mean age = 79.3 years, 65.5% female), 25% had MetS and 26.5% had CKD. Participants with CKD had a significantly higher prevalence of the MetS than individuals without CKD (34.4% vs. 24.3%). Binary logistic regression models showed that CKD was associated with MetS (OR = 1.72, 95%CI = 1.13–2.61). The association was unaltered by adjustment for hsCRP but altered by adjustment for HOMA-IR. As the number of MetS components increased the relative odds of CKD also increased. None of the individual components was independently associated with CKD.

**Conclusion:**

MetS is associated with CKD in non-diabetic older adults. Results showed that as the number of MetS components increased so did the odds for CKD. HOMA-IR seems to be in the casual pathway linking MetS to CKD.

## Introduction

About 47 million people in the US have the metabolic syndrome (MetS), 42% of whom are over the age of 70 [1]. Up to 20 million individuals have chronic kidney disease (CKD), 47% of whom are over the age of 70 [2,3]. Due to their increasing prevalence rates, both the MetS and CKD have emerged as significant public health problems [4].

The MetS is characterized by a constellation of CVD risk-factors and morbidities that include the presence of at least three of the following components: elevated fasting glucose, elevated triglycerides, elevated blood pressure, elevated waist circumference, and low high-density lipoprotein (HDL) cholesterol [1]. There is increasing evidence that the MetS is associated with incident and prevalent CKD, often defined by a poor glomerular filtration rate (GFR) below 60mL/min/1.73m [2,5]. Indeed individuals with CKD have higher prevalence rates of MetS components than individuals without CKD. Persons with CKD have higher rates of traditional and nontraditional cardiovascular risk factors, including insulin resistance and elevation of inflammatory markers [6, 7]. Insulin resistance is associated with diabetes, cerebrovascular disease, central obesity and hypertension [8–11]; it is characterized by a complex network of nutritional and metabolic changes that also include inflammation, oxidative stress, vitamin D deficiency, anemia, and malnutrition [12]. High sensitivity C-reactive protein (hsCRP), which is generally considered a marker of low-grade chronic inflammation, has also been associated with central obesity, atherosclerosis, hypertension and other components of the MetS [11, 13, 14]_._ It is thought that insulin resistance and inflammation are the main culprits in the development of CKD [5, 12, 15, 16].

Inflammation and insulin resistance have rarely been examined simultaneously as potential mediators of the influence of MetS on CKD [11, 17–21]. The Atherosclerosis Risk in Community (ARIC) Study, which consists of 15,000 individuals between the ages of 45 and 64 [20] and the Third National Health and Nutrition Examination Survey (NHANES), which includes over 6,000 adults over the age of 20 [17] as well as international studies (e.g. Ming et al.[21] investigated the CKD-MetS association in 15,987 Chinese individuals over the age of 20) have investigated the association between the MetS and CKD. However, these studies overlooked major risk factors, such as inflammation and insulin resistance that are also highly associated with CKD [17, 19] and that are potential pathways which may attenuate the MetS and CKD association. Exploring the interrelationships of the MetS, its components as well as insulin resistance and inflammation with CKD will help identify potential casual pathways. Research in community-dwelling elderly is also still lacking, mainly because most studies make use of lifespan datasets [11, 17, 19, 20]. Determining the associations of these metabolic risk factors with CKD in persons over age 70 is crucial to determining whether prevention strategies and treatment should be tailored to this older segment of the population.

In this study we aimed to explore the association between MetS and CKD. We also examined two potential casual pathways, inflammation and insulin resistance, to determine if they attenuate the associations between MetS and CKD. Our hypotheses were that the MetS is associated with CKD; insulin resistance and inflammation moderate this association but do not fully attenuate it.

We planned a set of secondary aims that included exploring the association between CKD and number of MetS components present (i.e. from 0 up to 4+), and exploring the association between CKD and individual components of the MetS to determine the number of components and the specific components that are associated with the MetS.

## Methods

This analysis was cross-sectional and was conducted within a subset of the Einstein Aging Study (EAS) cohort. EAS enrolls community-dwelling, English-speaking residents of Bronx county in New York who are 70 years or older. Participants were systematically recruited from the Health Care Financing Administration/Centers for Medicaid and Medicare Services rosters for Medicare-eligible persons who were 70 years or older between 1993 and 2004, and from New York City Board of Elections from 2004 onwards. Individuals are first mailed introductory letters about the study and research assistants then followed up by phoning to obtain oral consent and administer a brief screening interview. Participants were excluded if they had visual and/or auditory impairments that interfere with neuropsychological testing, psychiatric symptomatology that interferes with test completion, or a nonambulatory status. The study protocol was approved by the Albert Einstein College of Medicine Institutional Review Board. Written informed consent is obtained on their first clinical visit [22]. Individuals with dementia and diabetes at baseline status for eGFR were excluded from these analyses. A diagnosis of dementia was assigned at case conferences attended by a study neurologist, neuropsychologist, and a geriatric nurse clinician, using standardized criteria from the Diagnostic and Statistical Manual, Fourth Edition (DSM-IV) [23], which required impairment in memory plus at least 1 additional cognitive domain, accompanied by evidence of functional decline. History of diabetes was defined if the participant replied yes during the clinical interview to the question: “Did a doctor ever tell you that you have diabetes?” Baseline status here refers to the first wave of data for which participants have eGFR data.

### Demographic characteristics

We used age, gender, race, and years of education, current smoking status and alcohol intake in the past month as covariates in our models. These were collected from the clinical interview. Smoking and alcohol were included in the demographic covariates due to their known association with CKD.

### Definitions

We defined the metabolic syndrome as three of more of the following criteria, according to the National Cholesterol Education Program Adult Treatment Panel III [24]:

- Elevated waist circumference (≥102cm in men, and ≥88cm in women),
- Elevated triglycerides (≥150mg/dl),
- Reduced HDL cholesterol (<40mg/dL in men and <50mg/dL in women),
- High blood pressure (≥103/≥85mmHg or the use of antihypertensive medications)
- Elevated fasting glucose (≥100mg/dL).

We used high sensitivity C—reactive protein (hsCRP, mg/L) to assess inflammation. We used this as a continuous variable. We ran models using both raw units of hsCRP and its log transformation; since results remained similar after log transformation, we used the raw scores for our analyses.

Insulin resistance (IR) was defined using the homeostasis model assessment insulin resistance (HOMA-IR) equation [25]: [insulin (ulu/mL) x glucose (mmol/L) / 22.5]. We also used this as a continuous variable.

Chronic Kidney Disease (CKD) was defined as eGFR below 60 mL/min/1.73m^2^. We estimated eGFR in mL/min/1.73m^2^ using the Modification of Diet in Renal Disease (MDRD [26]) formula:

eGFR = 186 × Serum Creatinine^-1.154^ × Age^-0.203^ × [1.210 if Black] × [0.742 if Female]

The MDRD formula has been recommended for use in older people [27].

### Statistical analysis

Participants were divided into two subgroups; with and without metabolic syndrome. Differences in demographic and clinical characteristics were analyzed for the two subgroups. The independent t-test was used for continuous variables and the chi-square for categorical variables.

Participants were also divided according to with and without CKD to analyze means and prevalence rates of the metabolic syndrome and its individual components.

First, binary logistic regressions were used to assess the association of each of the metabolic syndrome components and CKD. The first model was adjusted for demographics, the second and third models were adjusted for hsCRP and HOMA-IR to find out if these mediated any associations. The fourth model was adjusted for demographics, hsCRP and HOMA-IR. Binary logistic regression models with the same adjustments were also used to assess the association between the number of components of the metabolic syndrome and CKD and lastly, to assess the association between individual components of the MetS and CKD. In these models we also included hsCRP and HOMA-IR to examine whether the association between number of, and individual metabolic syndrome components and CKD is modified by the presence of inflammation and/or insulin resistance. A Bonferroni correction [28] factor (with an adjusted *p* value of 0.01 for the five metabolic components (a = 0.05, five metabolic components) and for the number of metabolic components present (0, 1, 2, 3, or 4+; a = 0.05, five possible scenarios) was used to correct for Type I error in models that included multiple testing.

## Results

**Fig 1:**
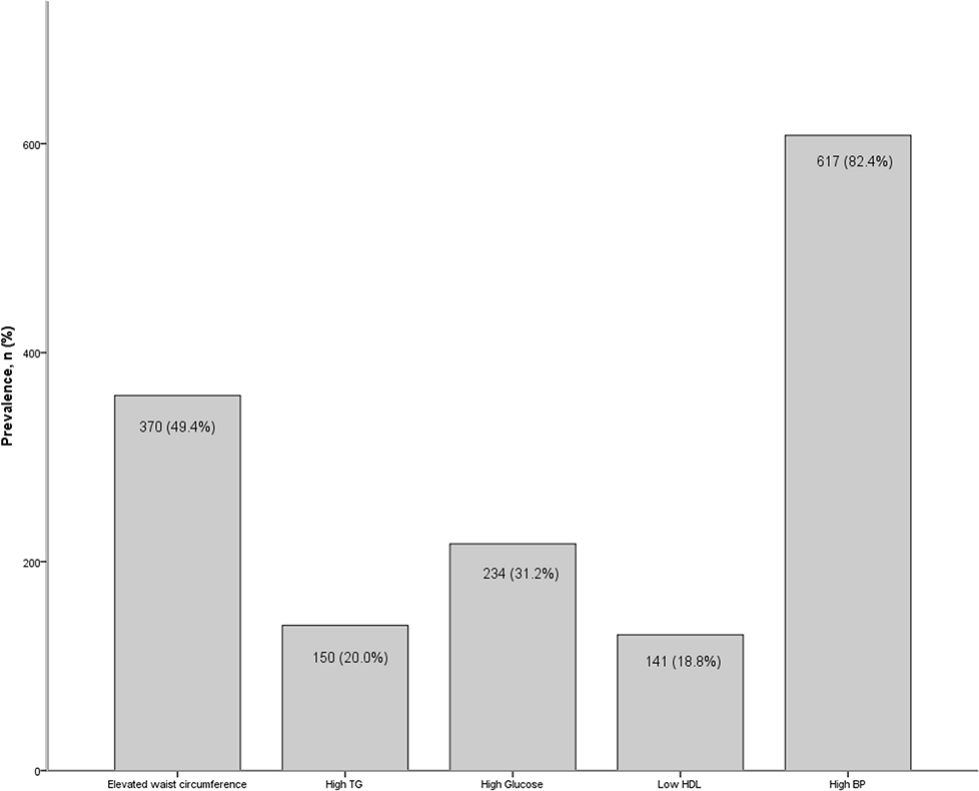
Proportion of participants with each component of the MetS. (A) TG = triglycerides. HDL = high-density lipoprotein cholesterol. BP = blood pressure.

**Fig 2:**
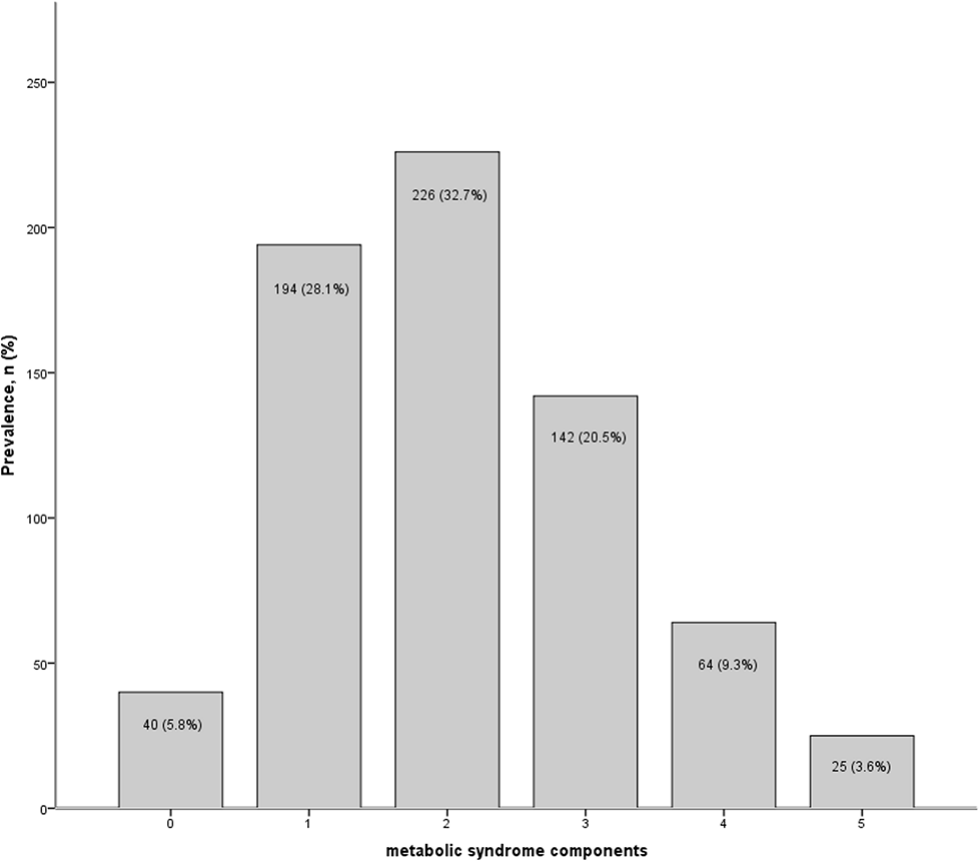
Proportion of study participants with specific number of components of the metabolic syndrome.

Table 1 lists the demographic and clinical characteristics of the population according to participants with and without the metabolic syndrome. Of the 616 participants included in the study, the mean age was 79.3 (SD = 5.5), 34.7% were male and 25.8% were black. 25% of the cohort met criteria for the metabolic syndrome and 26.5% were classified as having CKD. In contrast to participants without the metabolic syndrome, those with the metabolic syndrome had fewer years of education, significantly lower levels of eGFR and significantly higher prevalence rates of CKD. They also had higher mean level of hsCRP, and significantly higher levels of insulin resistance (Table 1). Fig 1 shows the most prevalent components of the metabolic syndrome, which were high blood pressure (n = 500, 81.2%) and elevated waist circumference (n = 276, 44.8%); and Fig 2 shows that most of these older individuals had at least 2 metabolic syndrome component risk factors present (n = 226, 32.7%). In total there were 151 (26.5%) participants defined as having CKD on the basis of eGFR. Participants classified as CKD had a significantly higher proportion of the metabolic syndrome than those without CKD (34.4% vs. 24.3%, *p* = .017). They were also on average significantly older (80.5 vs. 78.9, *p* = .004) and showed significantly higher HOMR-IR (8.29 vs. 5.42, *p* = .012) and higher mean hsCRP, although not significantly different from the no CKD group (4.04 vs. 3.39, *p* = 2.00). Table 2 shows the means and prevalence of individual metabolic syndrome components according to presence of CKD. Participants with CKD had significantly higher prevalence of elevated fasting glucose, elevated triglycerides and low HDL cholesterol than individuals without CKD.

**Table 1:** Demographic and clinical characteristics of the study participants with and without the metabolic syndrome.

	Metabolic syndrome		
	No	Yes	*P*
	(n = 416, 73%)	(n = 154, 27%)	
**Demographics**			
Age (y)	79.5 (5.6)	78.3 (5.3)	.017
Male (%)	164 (39.4)	49 (31.8)	.096
Non-Hispanic white (%)	286 (68.8)	108 (70.1)	.904
Non-Hispanic black (%)	107 (25.7)	40 (26.0)	
Other (%)	23 (5.5)	6 (3.9)	
Education (y)	14.4 (3.4)	14.1 (3.2)	.209
Current smokers (%)	17 (4.1)	7 (4.6)	.818
Alcohol consumption (%)	59 (14.2)	25 (16.2)	.547
**Clinical characteristics**			
CKD (%)	99 (23.8)	52 (33.8)	.017
eGFR (1.73/m^2^)	72.6 (18.7)	66.0 (17.8)	.000
Waist circumference (cm)	90.3 (11.3)	100.0 (10.8)	.000
Triglycerides (mg/dL)	93.0 (93.4)	164.1 (87.6)	.000
HDL cholesterol (mg/dL)	62.2 (15.4)	49.1 (12.4)	.000
Systolic blood pressure (mmHg)	136.0 (18.0)	137.6 (16.2)	.304
Diastolic blood pressure (mmHg)	77.3 (9.2)	77.5 (9.9)	.754
Fasting glucose (mg/dL)	89.7 (11.2)	105.7 (23.1)	.000
High sensitivity C-Reactive Protein (mg/L)	3.46 (5.7)	4.10 (4.3)	.243
HOMA insulin resistance	4.49 (5.9)	10.8 (14.5)	.000

*Note*. CKD = chronic kidney disease. eGFR = estimated glomerular filtration rate. HDL cholesterol = high density lipoprotein cholesterol. HOMA-IR = Homeostasis Model Assessment Insulin resistance.

**Table 2:** Prevalence of individual components of the metabolic syndrome in participants with and without chronic kidney disease.

Component		Chronic kidney disease			
		Missing, n (%)	No, n (%)	Yes, n (%)	*p* value
			419 (73.5)	151 (26.5)	
Fasting glucose	Mean (mg/dL)		92.3 (15.2)	98.6 (20.1)	.000
Elevated fasting glucose	Proportion ≥ 100 mg/dL	1 (0.1%)	91 (20.0)	48 (29.8)	.010
Waist circumference	Mean (cm)		92.3 (12.2)	94.8 (11.1)	.028
Elevated waist circumference	Proportion ≥102cm in men or ≥ 88cm in women	41 (5.3%)	194 (45.2)	82 (5.6)	.116
Triglycerides	Mean (mg/dL)		108.4 (60.5)	121.6 (64.5)	.020
Elevated triglycerides	Proportion ≥150mg/dL	1 (0.1%)	75 (16.5)	38 (23.6)	.046
HDL cholesterol	Mean (mmHg)		59.3 (16.0)	55.8 (14.9)	.018
Reduced HDL cholesterol	Proportion <40mg/dL in men or <50mg/dL in women	1 (0.1%)	69 (15.2)	37 (23.0)	.025
Systolic blood pressure	Mean (mmHg)		136.7 (17.4)	135.9 (18.1)	.636
Diastolic blood pressure	Mean (mmHg)		78.0 (8.8)	75.6 (10.8)	.015
Elevated blood pressure	Proportion ≥130/85 mmHg	50 (6.5%)	362 (85.2)	138 (91.4)	.053
Metabolic syndrome	Proportion with MetS	46 (7.5%)	102 (24.3)	52 (34.4)	.017
HOMA-IR	Mean		5.42 (7.29)	8.29 (13.3)	.012
High sensitivity C-Reactive protein	Mean (mg/L)		3.39 (5.1)	4.04 (5.4)	.200

*Note*. HOME-IR = Homeostasis Model Assessment Insulin Resistance. HDL = high-density lipoprotein.

Table 3 shows the odds ratio of CKD for metabolic syndrome defined as a dichotomous variable (presence of >3 components vs. < 3). Results showed that the MetS was associated with CKD independent of demographics and inflammation (OR = 1.83, CI = 1.16–2.88, *p* = .009); however, when HOMA-IR was entered in the model, the association between the MetS and CKD was attenuated, and HOMA-IR was significantly and independently associated with CKD (OR = 1.02, CI = 1.00–1.05, *p* = .038).

**Table 3:** Odds ratios for chronic kidney disease predicted by the metabolic syndrome with adjustments as described below.

	Odds Ratios (95% Confidence Intervals)			
	Model 1	Model 2	Model 3	Model 4
	Adjusted for demographics	Adjusted for demographics + hsCRP	Adjusted for demographics + HOMA-IR	Adjusted for demographics + hsCRP + HOMA-IR
Metabolic syndrome	1.72** (1.13–2.61)	1.83** (1.16–2.88)	1.45 (0.92–2.78)	1.48 (0.93–2.41)
hsCRP	-	1.03 (0.99–1.07)	-	1.04 (0.99–1.08)
HOMA-IR	-	-	1.02* (1.00–1.05)	1.02* (1.00–1.05)

*Note*. Demographics = age, gender, race, education, smoking and alcohol intake. hsCRP = high sensitivity C-reactive protein. IR = insulin resistance. HDL = high-density lipoprotein cholesterol.

*p>.05

**p < .01

In Table 4 we show the odds ratios of CKD associated with one, two, three, or four + components of the MetS compared to no components present. In the fully adjusted model, for individuals presenting with 4+ components, the OR for CKD was 7.65 (CI = 1.49–36.26, *p* = .015), independent of inflammation and HOMR-IR.

**Table 4:** Odds ratios for chronic kidney disease predicted by number of components of the metabolic syndrome with adjustments as described below.

	Odds Ratio (95% Confidence Interval)			
	Model 1	Model 2	Model 3	Model 4
	Adjusted for demographics	Adjusted for demographics + hsCRP	Adjusted for demographics + HOMA-IR	Adjusted for demographics + hsCRP + HOMA-IR
0 components	Ref.	Ref.	Ref.	Ref.
1 component	2.13 (0.77–5.89)	2.90 (0.82–10.31)	2.33 (0.76–7.12)	3.62 (0.80–16.35)
2 components	2.77 (1.01–7.57)	3.78 (1.07–13.39)	2.82 (0.93–8.56)	4.31 (0.96–19.48)
3 components	3.05 (1.07–8.74)	4.54 (1.23–16.78)	2.98 (0.94–9.48)	4.90 (1.04–22.99)
4+ components	6.18*** (2.03–18.82)	9.21** (2.03–36.81)	4.98* (1.45–17.10)	7.65* (1.49–39.26)
hsCRP	-	1.03 (0.99–1.07)	-	1.03 (0.99–1.08)
HOMA-IR	-	-	1.02 (1.00–1.04)	1.02 (1.00–1.04)

*Note*. Demographics = age, gender, race, education, smoking and alcohol intake. hsCRP = high sensitivity C-reactive protein. HOMA-IR = Homeostasis Model Assessment insulin resistance. HDL cholesterol = high-density lipoprotein cholesterol. A *p* value < .01 was considered statistically significant.

*p>.05

**p < .01

***p < .001

Table 5 shows the relative odds ratio of CKD associated with individual components of the metabolic syndrome. None of the MetS components were significantly associated with CKD. Elevated waist circumference was associated with CKD in models that included only demographics (OR = 1.44, CI = 1.01–2.03, *p* = .01, results not shown); however, once all other components were entered into the model this association was attenuated.

**Table 5:** Odds ratios for chronic kidney disease predicted by individual components of the metabolic syndrome with adjustments as described below.

	Odds ratio (95% confidence interval)			
	Model 1	Model 2	Model 3	Model 4
	Adjusted for demographics	Adjusted for demographics and hsCRP	Adjusted for demographics and HOMA-IR	Adjusted for demographics and hsCRP and HOMA-IR
Reduced HDL cholesterol	1.23 (0.73–2.08)	1.29 (0.73–2.29)	1.21 (0.70–2.10)	1.15 (0.63–2.07)
Elevated fasting glucose	1.71 (1.08–2.71)	1.70 (1.03–2.79)	1.42 (0.86–2.33)	1.38 (0.81–2.35)
Elevated waist circumference	1.33 (0.88–2.01)	1.45 (0.93–2.26)	1.31 (0.86–2.01)	1.38 (0.88–2.17)
Elevated triglycerides	1.15 (0.68–1.94)	1.13 (0.64–1.98)	1.03 (0.59–1.79)	1.03 (0.57–1.85)
Elevated blood pressure	1.70 (0.89–3.26)	2.01 (0.92–4.37)	1.58 (0.80–3.12)	2.07 (0.91–4.70)
hsCRP	-	1.03 (0.99–1.07)	-	1.03 (0.99–1.08)
HOMA-IR	-	-	1.02 (1.00–1.04)	1.02 (0.99–1.04)

*Note*. Demographics = age, gender, race, education, smoking and alcohol intake. hsCRP = high sensitivity C-reactive protein. IR = insulin resistance. HDL = high-density lipoprotein cholesterol. A *p* value of < .01 was considered statistically significant.

## Discussion

In this cross-sectional study, our aim was to characterize the associations of metabolic components with CKD in community-residing older adults using i) the MetS as a dichotomous variable (3+ vs <3 components) ii) the total number of components present (0 to 4+), and iii) individual components of the MetS. We also evaluated two potential mediators that may explain the association between the MetS and CKD: inflammation as measured by hsCRP, and insulin resistance as measured by HOMA-IR.

Individuals with MetS had higher odds for CKD; HOMA-IR attenuated the association between the MetS and CKD, though hsCRP did not. This suggests that the explanatory variance of MetS for CKD is better accounted for by HOMA-IR. hsCRP does not attenuate the association of MetS with CKD. Examining the total number of components present, we showed that as the numbers of MetS components increased so did the odds of CKD [F = (1, 1) = 13.82, p < .001]. Thus, as we added components starting from 0 and moving on to 4+ components of MetS (aim ii), odds for CKD also increased (OR = 7.98, CI = 1.57–40.60, p = .015 for 4+ components). Although not significant as a predictor of CKD, adding inflammation to the model (Table 4) increased the odds of CKD given components of the MetS (Models 2 and 4 in Table 4). Because hsCRP was not associated with CKD and did not attenuate the association between MetS and CKD (Tables 3 and 4), we suggest that inflammation as measured here may not be in the casual pathway linking the MetS to CKD. On the other hand, once HOMA-IR was entered in the models, MetS risk odds became weaker for CKD (Models 3 and 4 in Table 4). Thus, in line with previous research, our results also suggested that HOMA- IR may be in the casual pathway to CKD^7^ and that inflammation may not be associated with CKD-related events as some previous studies have suggested [29].

Our results are supported by previous studies. Results from the ARIC and the NHANES showed that the MetS was associated with CKD independent of diabetes and hypertension; individuals with up to five components present, as opposed to those without any components present, had an odds of 2.45 (CI = 1.32–4.54) of developing CKD in the ARIC [20] and a 5.85 odds ratio [confidence interval (CI) = 3.11–5.19] in the NHANES [17] Other studies also showed similar results. Ming et al.’s [21] study, showed that the odds ratio of CKD in individuals with MetS was 1.46 (CI = 1.15–1.86) as opposed to those without MetS; the odds still remained high (OR = 1.32, CI– 1.08–1.62) after excluding individuals with diabetes and controlling for demographics, physical activity, smoking and alcohol drinking. Similarly, Chen et al. [30] also explored this association in 15,160 Chinese adults between the ages of 35 and 74. Results also showed an independent increasing risk of CKD with higher prevalence of 5 MetS components (an OR of up to 2.72, CI = 1.50–4.39) compared to those without any components.

In our study, HOMA-IR was a strong predictor of CKD even though we excluded individuals with diabetes. HOMA-IR has played an important role in previous studies that excluded participants with diabetes too, such as in Chen et al. [17] and Kurella et al.[20] who found associations between HOMA-IR and CKD, and between CKD and MetS. Results from the NHANES data [17] showed that individuals with the highest insulin resistance had higher odds for CKD (OR = 2.65, CI = 1.25–5.62). However, this study did not explore other MetS components and limited the CKD risk-factors to just insulin-related variables, such as serum-insulin, HbA1C and insulin resistance. In a study on 2,380 Native Americans between the ages of 45 and 74 [31], the association between the MetS and CKD was stronger in individuals who developed diabetes during follow-up. HOMA-IR is increasingly becoming recognized as a nontraditional risk factor for CKD [12]. This measure is associated with worsening renal hemodynamics, sodium retention, overproduction of low-density lipoprotein cholesterol, and hypertriglyceridemia [12, 16, 32]. Insulin resistance is also associated with endothelial dysfunction attributed to structural arteriolar changes that lead to limited vasodilation and consequent reduction in endothelial nitric oxide synthase (eNOS) [33]. Metabolic syndrome and insulin resistance are risk factors for stroke and cardiovascular events [34, 35] and all-cause mortality [35, 36].

We therefore suggest future research to identify if the insulin resistance-MetS-kidney function association also extends to the brain. Since previous research has also found kidney-cognitive associations [20, 37, 38] and MetS-cognitive associations [39, 40], we encourage work that attempts to identify if these associations extend to brain and cognitive function using imaging techniques and neuropsychological testing. We also suggest investigating further the association between HOMA-IR and specific components of the MetS in stratified analysis analyzing individuals with CKD separately from those without. Lastly, we suggest that future research also investigate other non-traditional risk factors that may be associated with CKD with the aim of developing clinical trials specifically targeted at treating these risk factors. Although there is not much evidence available that shows that by preventing or treating the symptoms of MetS protects against, or reverses CKD, there are some insulin resistance treatments that aim at targeting metabolic acidosis, anemia, uremic toxins, vitamin D deficiency, malnutrition and even physical fitness [12, 16]. Although traditional risk factors, such as hypertension and diabetes, are high associates of CKD, they do not fully explain this condition. It seems that biomarkers of pathophisologies contributing to CKD may help in predicting and identify CKD in more precise terms.

A particular strength of this study was the defined age-group and the systemic community-based sample. The vast majority of studies use large age-ranges; limiting to one age-group may show specific associations to that particular group. Older adults are at higher risk of complications and comorbidity; learning what puts them at risk and what prevents it may lead to better prevention and treatment plans, longer independence, and better health and health-care management. Our study also had some limitations. Our sample size was relatively small when compared to other large databases. Furthermore, our participants were relatively healthy compared to the rest of their age group–this is because we excluded individuals with diabetes and dementia, and also because our study only includes independent community-dwelling individuals, thus excluding anyone who may be hospitalized or living in a nursing home and therefore more likely to have disease. This may be evident in the proportion that met the criteria for MetS in our sample (25%), (in the introduction we state that 42% of older adults have MetS^1^). Although we excluded participants with diabetes, this was only based on self-report, so it may be possible that we included some participants with undiagnosed diabetes. We only had available a single marker of inflammation (hsCRP). Lastly, this study was cross-sectional, aiming to identify associations and risk factors; however, it is encouraged that longitudinal work is followed up on these variables to find out if these associations become stronger over time, and if MetS and insulin resistance predict incident CKD.

## Conclusion

In conclusion, results from this study showed that i) the single definition of the MetS does not survive adjustment for HOMA-IR; ii) MetS studied by number of components present is a better measure of the MetS than by it single definition; iii) single components of the MetS on their own are not significant independent risk factors for CKD; and that iv) hsCRP per se may not be of key importance in the prevalence of CKD, but rather HOMA-IR seems to have a more prominent role. The association between insulin resistance and CKD should be further investigated.
